# Left-Handedness

**Published:** 1909-01-09

**Authors:** 


### LEFT-HANDEDNESS.

A good many anatomists and physiologists have from time to time attacked the problem as to how and why the majority of the human species are right-handed, right-eyed, and right-footed, whereas a small percentage prefer the use of the organs of the left side; and a good many ingenious hypotheses have been hatched out in explanation of this curious trait. No single one of these conjectures—for they are little more—has obtained acceptance over all others, though one or two present a certain show of inherent probability. However, a psychologist has recently published what he calls an inquiry into the causes of the anomaly of left-handedness, in which he discards everything hitherto suggested, and falls back on a wholly imaginary and inexplicable "positiveness" of the right side, which he contrasts with the "negativeness" of the left side by the analogy of "electric force or current." Into the muddle of confused verbiage and abysmal ignorance, wherewith. this explanation which explains nothing is propounded, it profits little to follow him: the materialism of the scientist, for which he professes considerable contempt, seems a safer and more solid foundation for attempts to guess the riddle. But some of the solutions already put forward are really worthy of a little more attention than they receive at the hands of their psychologist critic. That which is perhaps the best known, and has the greatest appearance of reason, was advocated years ago by Dr. Pye-Smith. According to this hypothesis it is the eccentric position of the heart which accounts for the greater dexterity—this word itself shows how ancient and ingrained is right-handedness—of the right upper limb. It was pointed out that, supposing the human race to be ambidextrous when the first shield was invented, those who used the spear in the right hand and the shield in the left would be less likely to sustain mortal wounds in battle than those who adopted the reverse plan. Hence in process of time natural selection would create a race of men who preferred the right hand to the left for especially skilled work. There is nothing in this conception in any way inconsistent with the Darwinian theory of evolution: nay, rather, in respect of few qualities would beneficial variations more quickly become established, for immediate death itself is the penalty of clinging to left-handed spear-wielding. The respectable percentage of the naturally left-handed at the present day would also be explained by the (geologically) recent date at which shields have been invented. Granted the premise that the left side of the trunk is the more vulnerable, because of the situation of the heart, this guess is not one to be lightly pooh-poohed. The psychologist dismisses it airily on three grounds, all totally invalid and all clear proof that he cannot ever have read the Origin of Species. So, too, he dismisses the vein theory of Dr. Barclay, which supposed the very slight obstruction to the main veins of the extremities on the left side by their relation to some of the large arteries to be sufficient to hinder, infinitesimally but effectively, the relative nutrition of the left side. There is one remark to be made about this hypothesis, and it is that left-handed persons should be proved to possess abnormal arterial or venous trunks before it can be accepted; but this is not why the psychologist will have none of it. Then he proceeds to fall foul of Dr. Bastian on a question of the brain, with which unprofitable task we will leave him.

